# Tandem T:Slim X2 Insulin Pump Use in Clinical Practice Among Pregnant Individuals With Type 1 Diabetes: A Retrospective Observational Cohort Study

**DOI:** 10.7759/cureus.52369

**Published:** 2024-01-16

**Authors:** Neeharika Nandam, Stephen Thung, Kartik K Venkatesh, Steven Gabbe, Jianing Ma, Jing Peng, Kathleen Dungan, Elizabeth O Buschur

**Affiliations:** 1 Department of Endocrinology, Diabetes, and Metabolism, Cleveland Clinic, Cleveland, USA; 2 Division of Maternal Fetal-Medicine, Department of Obstetrics, Gynecology, and Reproductive Sciences, Yale School of Medicine, Bridgeport, USA; 3 Department of Obstetrics and Gynecology, Division of Maternal-Fetal Medicine, Ohio State University Wexner Medical Center, Columbus, USA; 4 Center for Biostatistics, Ohio State University Wexner Medical Center, Columbus, USA; 5 Division of Endocrinology, Diabetes, and Metabolism, Ohio State University Wexner Medical Center, Columbus, USA

**Keywords:** time in range (tir), type 1 diabetes mellitus (t1d), pregnancy, insulin pump, hybrid closed loop, automated insulin delivery

## Abstract

Background: Insulin pump use is increasing in frequency among pregnant individuals with type 1 diabetes (T1D). Automated insulin delivery (AID) technologies have not been studied extensively in pregnancy.

Method: We present a retrospective case series of eight individuals with T1D who used the Tandem t:slim X2 insulin pump (Tandem Diabetes Care, Inc., CA, USA) during pregnancy. Weekly continuous glucose monitor and insulin pump data were analyzed from electronic medical records and data-sharing portals. Safety, glycemic control, and pregnancy outcomes were examined with both the control IQ (CIQ) and basal IQ (BIQ) algorithms.

Results: Six CIQ and two BIQ users were studied. The mean glycated hemoglobin (A1C) during pregnancy was 6.1%, and the average time in pregnancy-recommended glycemic range (TIR; 63-140mg/dL) was 67.9%. There were no instances of diabetic ketoacidosis or severe hypoglycemia. CIQ users had a higher mean sensor glucose (127.6 mg/dL) compared to BIQ participants (118.4 mg/dL). However, the average time below range (<63 mg/dL) was 6.1% in BIQ participants compared to 1.5% in CIQ participants. CIQ participants used several strategies to achieve glycemic targets, including daytime use of sleep activity. An increased basal-to-bolus insulin ratio was negatively correlated with TIR (r=-0.415).

Conclusions: Tandem t:slim X2 insulin pumps were safely used during pregnancy in eight individuals with T1D, with variable success in achieving recommended glycemic targets. Further research is needed to understand differences in CIQ and BIQ use in pregnancy. AID device manufacturers must additionally develop further methods to target lower glucose for pregnant users.

## Introduction

Type 1 diabetes (T1D) is associated with an increased rate of adverse pregnancy outcomes, such as preeclampsia [1]. Pregnant individuals with T1D are at greater risk of developing hypoglycemia due to the need for strict glycemic control to prevent adverse outcomes [2]. They are additionally at risk of severe hyperglycemia and diabetic ketoacidosis (DKA) due to greater insulin resistance in the second and third trimesters [2].

Nearly two in five individuals with T1D in the United States use an insulin pump, which has been shown in non-pregnant individuals to improve glycated hemoglobin (A1C) and quality of life [2]. Insulin pump manufacturers have developed predictive technologies for automated insulin delivery (AID) in response to continuous glucose monitoring (CGM) values [3,4]. These systems decrease rates of hypoglycemia and increase time in the goal glycemic range compared to an insulin pump and CGM use without a predictive algorithm (sensor-augmented pump (SAP) therapy) [3,4].

AID technologies’ safety and efficacy have not been studied extensively in pregnant individuals with T1D, and they are not approved in the United States for use during pregnancy [5]. Furthermore, these products use algorithms with glucose targets that are higher than the tight glycemic range recommended in pregnancy [5]. Pregnant individuals with T1D who wish to use their AID insulin pumps during pregnancy must therefore do so on an off-label basis [5]. A study in Poland included pregnant individuals with T1D using CGM either with SAP therapy (n=15) or with automated predictive low glucose suspension or suspension on low glucose (n=13) using the Medtronic 640G insulin pump (Medtronic, MN, USA) [6], which is not available in the United States. This system is most similar to the basal IQ (BIQ) group in this study. This study found improved HbA1c in the third trimester between insulin pump users with CGM and those with pumps without CGM or on multiple daily injections [6].

There is a need for further research on the safety, glycemic control, and relative risk of adverse pregnancy outcomes with the use of newer insulin pump technologies in pregnancy in order to guide clinical management. The Cambridge artificial pancreas system (CamDiab Ltd., Cambridge, England) significantly decreased overnight hypoglycemia risk compared to SAP in pregnant individuals with T1D [7,8]. Additional case series studies have demonstrated the safety of the Medtronic 670G and 780G pumps in pregnancy (Medtronic, MN, USA) [9-11]. Other commonly used AID pumps, including the Tandem t:slim X2 (Tandem Diabetes Care, Inc., CA, USA) and Omnipod 5 (Insulet Corporation, MA, USA), have not been studied widely in pregnant individuals [12].

The Tandem t:slim X2 insulin pump offers three modes of operation: a predictive low-glucose suspend system called BIQ, a hybrid closed loop system called control IQ (CIQ), or SAP [13]. BIQ suspends insulin delivery if the CGM glucose is predicted to fall below 80 mg/dL in the next 30 minutes or if the value is already below 70 mg/dL [13]. In addition to reducing and suspending insulin delivery for hypoglycemia, the CIQ algorithm adjusts basal insulin delivery by a multiplier and delivers automated correction boluses for predicted or actual hyperglycemia [13]. CIQ targets glucose between 112.5 and 160 mg/dL at rest, 140-160 mg/dL during exercise activity, and 112.5-120 mg/dL with sleep activity, which modulates basal insulin without delivering automated boluses [13]. While BIQ or CIQ users can revert to SAP, the BIQ and CIQ algorithms are not interchangeable with each other [13].

Neither algorithm currently has strict enough glucose targets to meet glycemic recommendations for pregnancy [5]. The International Consensus on Use of Continuous Glucose Monitoring recommends a time in pregnancy range (TIR; 63-140 mg/dL) over 70%, time above range (TAR) less than 25%, and time below range (TBR) less than 5%, which differ from recommendations in non-pregnant individuals [14]. The Tandem t:slim X2 insulin pump integrates with the Dexcom G6 CGM as a glucose sensor (Dexcom, Inc., CA, USA) [13]. The use of Dexcom G6 is off-label for pregnant individuals in the United States, though it is approved in Europe [15]. The US Food and Drug Administration recently approved the Dexcom G7 as well as the Abbott FreeStyle Libre 2 and Libre 3 CGMs (Abbott, IL, USA) for use in pregnancy [15,16].

The objective of the current study was to examine the safety and efficacy of the Tandem t:slim X2 insulin pump in pregnant individuals with T1D in real-world clinical practice.

## Materials and methods

Study setting

We conducted a retrospective observational case series. Data were obtained through chart review of the electronic medical record (EMR); Dexcom Clarity, an online portal of CGM data from Dexcom, Inc.; and t:connect, an online portal of insulin pump data operated by Tandem Diabetes Care, Inc. This study was approved by the Ohio State University (OSU) Biomedical Sciences Institutional Review Board (approval no. 2022H0126), which waived the requirement for informed consent due to the retrospective nature of this study and the use of de-identified data. All study procedures followed the standards of the US Federal Policy for the Protection of Human Subjects.

OSU is a large academic medical center with multiple endocrinology and maternal-fetal medicine (MFM) providers that treat patients with diabetes during pregnancy. Patients are advised that the Dexcom G6 CGM and Tandem t:slim X2 insulin pumps are not approved for pregnancy and that they should perform adjunctive self-monitored blood glucose testing per current guidelines [16,17]. A goal of fasting glucose <95 mg/dL, one-hour postprandial glucose <140 mg/dL, and two-hour postprandial glucose <120 mg/dL are advised.

Remotely shared data from Dexcom Clarity and/or t:connect is reviewed weekly, and recommendations for pump adjustments are provided via electronic portal messaging. If unable to share data with t:connect, patients are asked to send their current pump settings electronically. Patients additionally have appointments for diabetes management on average once a month during pregnancy, either in-person or via synchronous telemedicine visits. An A1C is ordered once per trimester. Patients have access to diabetes educators and registered dietitians as needed. Additional obstetric management is conducted per current guidelines [16-18].

Study participants

Individuals who followed for diabetes care in pregnancy with OSU endocrinology or OSU MFM were screened through a review of the EMR. Inclusion criteria included being age 18-45 at the initial pregnancy visit, having T1D for over one year, using the Tandem t:slim X2 insulin pump throughout gestation, having shared data with either clinic t:connect and/or Dexcom Clarity accounts, and delivering between June 1, 2018, and May 5, 2022, at OSU or an outside hospital with shared EMR. Individuals were excluded if they had a non-viable pregnancy between 24 0/7 and 31 6/7 weeks gestation, used non-insulin diabetes medications during pregnancy, or did not meet the inclusion criteria. Out of 22 patients screened, eight patients were included who met all study criteria. The most common reason for exclusion was not being connected to clinic t:connect or Dexcom Clarity accounts.

Data collection and analysis

We analyzed monthly records of individual CGM readings to determine the percentage of time spent within, above, and below the pregnancy goal glycemic range (63-140 mg/dL). CGM values were extracted from remotely shared t:connect accounts or Dexcom Clarity if t:connect shared data was unavailable.

Additional glycemic variables were obtained from Dexcom Clarity CGM downloads in the EMR, including CGM time in use, average sensor glucose, coefficient of variation, standard deviation, and glucose management indicator (GMI) values [19]. If CGM downloads were unavailable, average sensor glucose from pump downloads was converted to an estimated A1C to use in lieu of the GMI [20]. Insulin pump downloads from the EMR were analyzed to determine the average total daily dose (TDD) of insulin, basal and bolus insulin percentages, CIQ or BIQ usage, sleep activity, and exercise activity use.

Demographic factors, comorbidities, A1C, and incidence of DKA and severe hypoglycemia (defined as hypoglycemia requiring third-party assistance) were obtained through chart review. Individual insulin pump setting changes were also counted, including changes to carbohydrate ratios and insulin type. The following adverse pregnancy outcomes were analyzed through chart review of clinic notes and notes from the admission for delivery: mode of delivery, preterm delivery <37 weeks, preeclampsia, macrosomia, large or small for gestational age, neonatal intensive care unit (NICU) admission, fetal loss, post-partum hemorrhage, and maternal death. Neonatal records were not accessed.

Study data were collected and stored using REDCap electronic data capture tools hosted by the Center for Clinical and Translational Science at OSU [21,22]. R version 4.1.1 (The R Foundation) and Microsoft Excel version 16.66.1 (Microsoft Corporation, WA, USA) were used for statistical analyses [23,24]. The mean and standard deviation are described for continuous variables. The number observed and percentage of the total sample are reported for categorical variables. Due to the low sample size, statistical significance analysis was not done.

## Results

Eight individuals were included in this case series, all of whom used the Tandem t:slim X2 insulin pump prior to pregnancy. Six used CIQ, and two used BIQ. At the initial pregnancy visit, the average age was 30.6 ± 6.1 years, and the mean gestational age was 9.3 ± 4.1 weeks. The average duration of T1D at the initial visit was 12.5 ± 5.8 years, the mean A1C was 6.7% ± 1.0%, and the mean TDD of insulin was 56.1 ± 29.4 units per day. Patient characteristics and comorbidities are summarized in Table 1. There were no instances of smoking, alcohol, or substance use during pregnancy.

**Table 1:** Baseline characteristics of the study participants CIQ: control IQ; BIQ: basal IQ; SD: standard deviation; T1D: type 1 diabetes; A1C: glycated hemoglobin; N/A: not applicable

Characteristic	Overall (n=8)	CIQ (n=6)	BIQ (n=2)
Race			
White	8 (100%)	6 (100%)	2 (100%)
Non-White	0 (0%)	0 (0%)	0 (0%)
Hispanic or Latino	2 (25%)	2 (33%)	0 (0%)
CIQ or BIQ use	N/A	6 (75%)	2 (25%)
	Overall mean ± SD	CIQ mean ± SD	BIQ mean ± SD
Age at initial visit (years)	30.6 ± 6.1	32.0 ± 5.9	26.5 ± 6.4
Gestational age at initial visit (weeks)	9.3 ± 4.1	8.8 ± 4.8	10.5 ± 0.7
Weight at initial visit (kg)	75.4 ± 17.0	72.0 ± 17.9	85.5 ± 11.9
BMI at initial visit (kg/m2)	27.6 ± 6.2	26.6 ± 6.2	30.7 ± 6.8
Duration of T1D diagnosis (years)	12.5 ± 5.8	11.2 ± 4.6	16.5 ± 9.2
Baseline A1C (%)	6.7 ± 1.0	6.4 ± 0.9	7.5 ± 0.7
Diabetes complications - n (%)			
Retinopathy	2 (25%)	1 (17%)	1 (50%)
Neuropathy	2 (25%)	1 (17%)	1 (50%)
Nephropathy	0 (0%)	0 (0%)	0 (0%)
Cardiovascular disease	0 (0%)	0 (0%)	0 (0%)
Hypothyroidism - n (%)	2 (25%)	1 (17%)	1 (50%)
Adrenal insufficiency - n (%)	1 (13%)	1 (17%)	0 (0%)
Chronic hypertension - n (%)	1 (13%)	0 (0%)	1 (50%)
Hyperlipidemia - n (%)	5 (63%)	4 (67%)	1 (50%)
Gravidity	2.5 ± 1.8	2.7 ± 2.0	2.0 ± 1.4
Parity			
Term births	0.6 ± 1.1	0.8 ± 1.2	0.0 ± 0
Premature births	0.3 ± 0.7	0.3 ± 0.8	0.0 ± 0
Abortions	0.8 ± 1.0	0.5 ± 0.5	1.5 ± 2.1
Living children	0.8 ± 1.2	1.0 ± 1.3	0.0 ± 0
Total daily dose of insulin at initial visit (units)	56.1 ± 29.4	49.3 ± 31.4	76.4 ± 4.1
Basal insulin percentage at initial visit (%)	53.9 ± 10.6	52.5 ± 11.9	58.0 ± 5.7
Bolus insulin percentage at initial visit (%)	46.1 ± 10.6	47.5 ± 11.9	42.0 ± 5.7

The mean A1C during pregnancy was 6.1% ± 0.8%, with CIQ users averaging 6.1% ± 0.7% and BIQ users averaging 6.0% ± 1.1%. The mean sensor glucose was 127.6 ± 16.8 mg/dL in CIQ and 118.4 ± 16.4 mg/dL in BIQ participants. CIQ participants had an average TIR of 67.9% ± 15.7%, TAR of 30.6% ± 16.0%, and TBR of 1.5% ± 1.4%. BIQ participants averaged 68.1% ± 12.0% TIR, 25.9% ± 12.3% TAR, and 6.1% ± 2.4% TBR. CIQ users provided 14.5 ± 13.7 CGM downloads and 23.8 ± 7.6 insulin pump downloads and had 70.7 ± 24.9 changes made to pump settings. BIQ users provided 5.0 ± 1.4 CGM downloads and 7.5 ± 7.8 pump downloads and underwent 33.5 ± 34.6 pump setting changes (Table 2).

**Table 2:** Mean glycemic and insulin pump metrics during pregnancy † BIQ and CIQ algorithms are not interchangeable with each other; however, each algorithm can be selectively turned off to use as a sensor-augmented pump. ‡ Sleep activity and exercise activity are only available as features in CIQ pumps. SD: standard deviation; A1C: glycated hemoglobin; CGM: continuous glucose monitor; GMI: glucose management indicator; CIQ: control IQ; BIQ: basal IQ; N/A: not applicable

Metric	Overall mean ± SD	CIQ mean ± SD	BIQ mean ± SD
Average A1C (%)	6.1 ± 0.8	6.1 ± 0.7	6.0 ± 1.1
Sensor glucose (mg/dL)	126.5 ± 17.0	127.6 ± 16.8	118.4 ± 16.4
Total daily dose of insulin (units)	73.1 ± 40.5	71.5 ± 43.2	82.2 ± 15.2
Basal insulin (%)	47.0 ± 13.1	44.9 ± 12.4	60.6 ± 8.7
Bolus insulin (%)	53.0 ± 13.2	55.1 ± 12.5	39.4 ± 8.7
Time in target glucose range 63-140 mg/dL (%)	67.9 ± 14.8	67.9 ± 15.7	68.1 ± 12.0
Time above range >140 mg/dL (%)	29.4 ± 15.2	30.6 ± 16.0	25.9 ± 12.3
Time below range <63 mg/dL (%)	2.6 ± 2.5	1.5 ± 1.4	6.1 ± 2.4
CGM coefficient of variation (%)	28.7 ± 6.7	28.1 ± 6.6	35.6 ± 4.8
CGM standard deviation (mg/dL)	36.7 ± 12.2	36.6 ± 12.4	37.9 ± 10.0
GMI (%)	6.2 ± 0.4	6.2 ± 0.4	5.9 ± 0.6
Time CGM active (%)	95.4 ± 10.4	95.7 ± 10.5	92.9 ± 10.2
Time CIQ active (%)	N/A^†^	87.7 ± 17.2	N/A^†^
Time BIQ active (%)	N/A^†^	N/A^†^	75.1 ± 14.9
Time CIQ or BIQ turned off^†^ (%)	5.6 ± 15.2	6.1 ± 15.9	0.0 ± 0
Insulin Pump and CGM Summary			
Total # of CGM downloads	12.1 ± 12.4	14.5 ± 13.7	5.0 ± 1.4
Total # of pump downloads	19.8 ± 10.3	23.8 ± 7.6	7.5 ± 7.8
Total # of individual pump setting changes	61.4 ± 30.2	70.7 ± 24.9	33.5 ± 34.6
Average sleep time (HH:MM) per 24 hours	N/A^‡^	11:12 ± 8:04	N/A^‡^
Average # of exercise events per pump download	N/A^‡^	0.3 ± 1.2	N/A^‡^

The average TDD of insulin increased by 96.1%, from 51.1 ± 27.1 units in the first trimester to 100.2 ± 53.1 units in the third trimester. The CIQ group required 99.7 ± 56.8 units in the third trimester (42.1% ± 11.3% basal insulin and 57.9% ± 11.2% bolus insulin). One of the two participants in the BIQ group was lost to follow-up partway through the third trimester, and their pump data was unavailable. The other BIQ participant required an average TDD of insulin of 103.5 units in the third trimester, with 66.2% as basal insulin and 33.8% as bolus insulin (Table 3).

**Table 3:** Glycemic and insulin pump metrics per trimester † Standard deviation not provided due to few available insulin pumps and CGM downloads during this trimester in the BIQ group (<3 per participant). ‡ Third trimester results for the BIQ group reflect insulin pump and CGM data for Participant 4 and only CGM data for Participant 7. Participant 7 was lost to follow-up during the third trimester and had no pump data available to view during this time in the electronic medical record. SD: standard deviation; A1C: glycated hemoglobin; CGM: continuous glucose monitor; GMI: glucose management indicator; CIQ: control IQ; BIQ: basal IQ; N/A: not applicable

Metrics	Overall mean ± SD			CIQ mean ± SD			BIQ mean ± SD		
	1st	2nd	3rd	1st	2nd	3rd	1st^†^	2nd	3rd^†^^,^^‡^
A1C (% )	6.5 ± 0.9	5.8 ± 0.6	5.9 ± 0.6	6.3 ± 0.8	5.8 ± 0.6	6.1 ± 0.5	7.5	5.5 ± 0.5	5.4
Sensor glucose (mg/dL)	120.5 ± 15.6	126.9 ± 17.7	130.5 ± 15.9	120.4 ± 15.8	128.7 ± 17.3	131.4 ± 15.5	121.3	117.5 ± 17.2	119.0
Time in pregnancy target glucose range 63-140 mg/dL (%)	71.6 ± 14.7	66.4 ± 16.2	67.1 ± 12.7	73.0 ± 15.5	66.6 ± 17.3	65.3 ± 12.6	67.3	65.7 ± 11.8	71.0
Time above range >140 mg/dL (%)	25.9 ±14.5	31.0 ± 16.6	30.1 ± 14.1	24.9 ± 15.3	31.9 ± 17.8	33.7 ± 13.3	29.1	27.2 ± 11.1	22.1
Time below range <63 mg/dL (%)	2.5 ± 1.7	2.6 ± 2.8	2.8 ± 2.9	2.1 ± 1.8	1.5 ± 1.3	1.0 ± 0.8	3.6	7.2 ± 2.8	6.8
CGM coefficient of variation (%)	27.0 ± 8.8	29.4 ± 6.6	29.0 ± 4.2	26.2 ± 8.4	28.6 ± 6.5	29.1 ± 4.3	41.5	35.9 ± 3.4	28.2
CGM standard deviation (mg/dL)	33.1 ± 14.3	37.6 ± 12.2	38.2 ± 9.8	31.9 ± 13.5	37.8 ± 12.9	38.7 ± 9.7	58.0	35.9 ± 3.4	28.0
GMI (%)	6.1 ± 0.4	6.2 ± 0.5	6.3 ± 0.4	6.1 ± 0.4	6.2 ± 0.5	6.3 ± 0.4	6.7	5.9 ± 0.4	5.8
Time CGM active (%)	97.6 ± 5.1	95.3 ± 7.3	93.9 ± 16.8	97.7 ± 5.1	95.9 ± 6.2	93.8 ± 17.3	94.4	92.4 ± 11.4	95.3
Time CIQ active (%)	N/A	N/A	N/A	95.7 ± 5.8	87.9 ± 17.8	81.1 ± 19.7	N/A	N/A	N/A
Time BIQ active (%)	N/A	N/A	N/A	N/A	N/A	N/A	82.0	77.3 ± 13.1	61.5
Time CIQ or BIQ turned off (%)	0.0 ± 0	4.4 ± 13.6	12.2 ± 21.0	0.0 ± 0	5.0 ± 14.5	12.9 ± 21.4	0.0	0.0 ± 0	0.0
Average sleep time (HH:MM) per 24 hours	N/A	N/A	N/A	9:48 ± 6:13	12:24 ± 8:57	10:18 ± 7:42	N/A	N/A	N/A
Total daily dose of insulin (units)	51.1 ± 27.1	72.1 ± 32.0	100.2 ± 53.1	48.8 ± 27.8	70.3 ± 34.6	99.7 ± 56.8	68.6	79.8 ± 10.3	103.5
Basal insulin (%)	48.4 ± 12.4	46.2 ± 12.9	47.1 ± 14.3	47.6 ± 12.5	44.8 ± 12.8	42.1 ± 11.3	57.7	55.9 ± 9.1	66.2
Bolus insulin (%)	51.6 ± 12.4	53.9 ± 13.1	52.8 ± 14.2	53.9 ± 12.4	55.3 ± 13.0	57.9 ± 11.2	42.3	44.1 ± 9.1	33.8

Exercise activity use was only seen in two CIQ users. Sleep activity duration did not consistently increase or decrease across trimesters (Table 3). However, multiple participants had sleep activity durations over 12 hours, implying daytime use of this feature. SAP use, indicated as the percentage of time CIQ or BIQ was turned off, was seen in two participants. Participant 3 had 37.0% SAP use in the second trimester and subsequently reverted to continuous CIQ use. Participant 5 started using SAP overnight in the third trimester, for an average duration of 45.2%.

**Figure 1:**
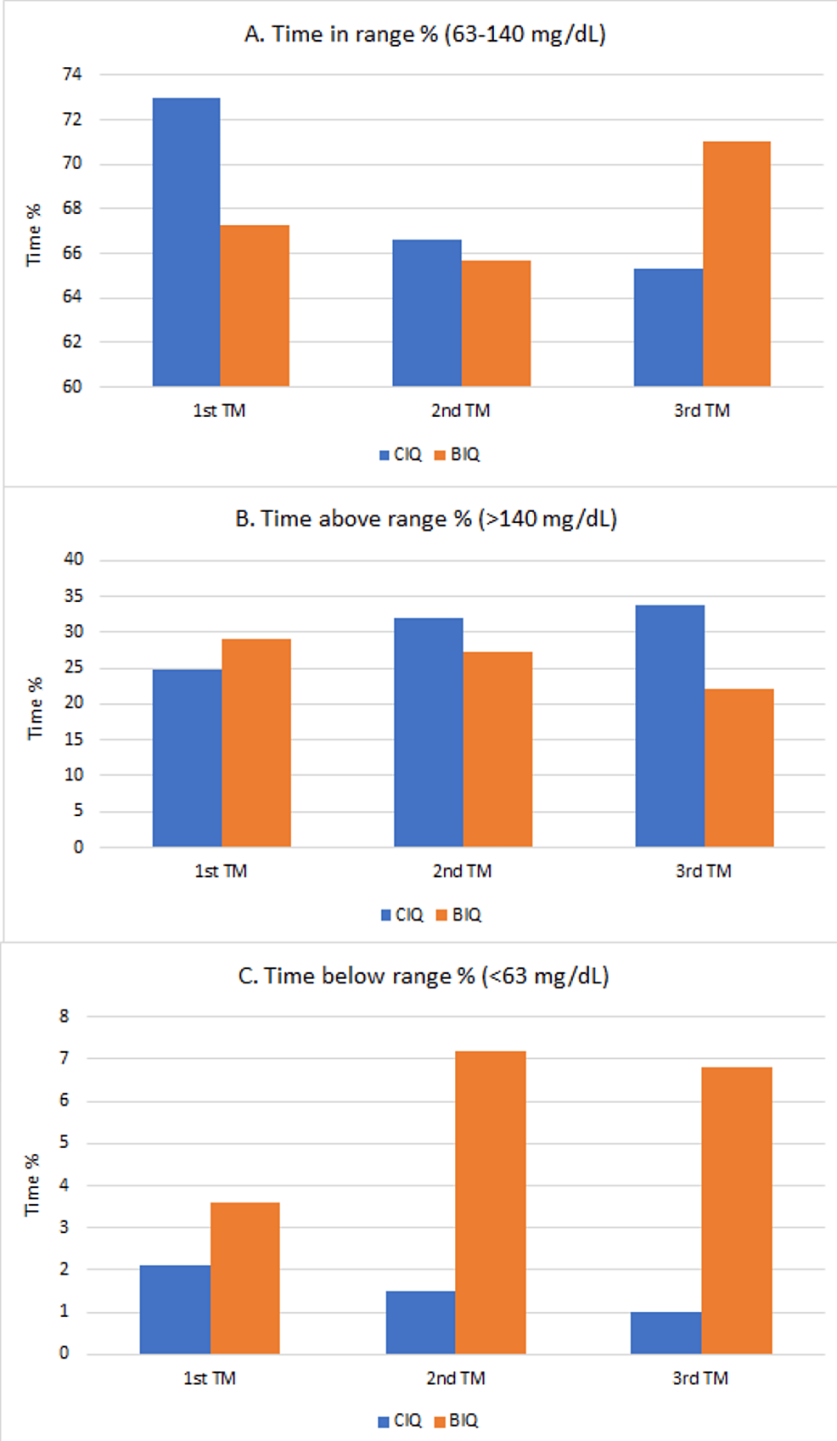
Comparison of pregnancy-specific glycemic ranges per trimester Current recommendations are to maintain a TIR above 70%, a TAR less than 25%, and a TBR less than 5% during pregnancy. Panel A demonstrates that the CIQ group met the recommended duration of higher than 70% in the range in the first trimester but fell below 70% in the second and third trimesters. The BIQ group was able to exceed 70% TIR in the third trimester. Panel B shows the BIQ group was above 25% TAR in the first and second trimesters and below 25% in the third trimester. The CIQ group in comparison had greater than 25% TAR in the second and third trimesters. Panel C demonstrates that on average, the BIQ group spent a greater amount of TBR, with this difference becoming more pronounced in the second and third trimesters. TM: trimester; CIQ: control IQ; BIQ: basal IQ; TIR: time in range; TAR: time above range; TBR: time below range

**Figure 2:**
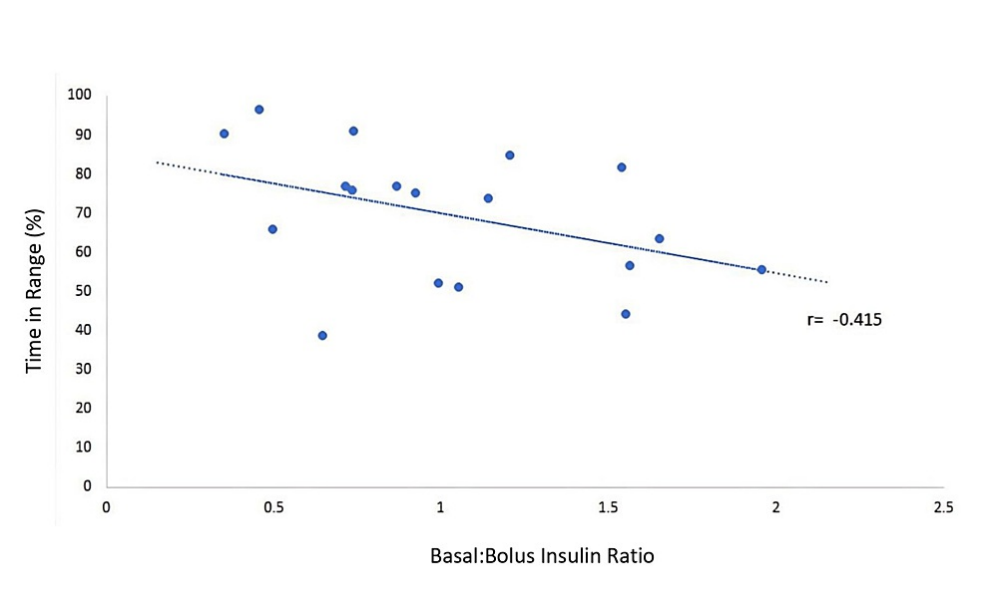
Basal and bolus insulin usage and time in range This figure demonstrates the ratio of basal insulin to bolus insulin percentage (x-axis) compared to the percentage of time in the range (63-140 mg/dL; y-axis) during that same period. As the ratio of basal insulin to bolus insulin increased, a negative correlation was seen with time in range (r=-0.415).

From the first to the third trimesters, average TIR decreased from 71.6% ± 14.7% to 67.1% ± 12.7%, TAR increased from 24.9% ± 14.5% to 30.1% ± 14.1%, and TBR increased from 2.5% ± 1.7% to 2.8% ± 2.9% (Table 3). CIQ users had a higher TAR in the second and third trimesters, and BIQ users had a higher TBR throughout pregnancy (Figure 1). Comparing the second trimester, which had the most data available, CIQ users averaged 31.9% ± 17.8% TAR compared to 27.2% ± 11.1% in BIQ participants. BIQ users averaged 7.2% ± 2.8% TBR, compared to 1.5% ± 1.3% in the CIQ group (Table 3). Lower TIR values were seen in participants with higher basal insulin usage, demonstrating a negative correlation between the basal-to-bolus insulin ratio and TIR (r=-0.415; Figure 2).

There were no instances of fetal or maternal death, severe hypoglycemia, DKA, or postpartum hemorrhage. Half of the cohort had pre-eclampsia (Table 4). Half of the participants had early deliveries prior to 37 weeks. The mean gestational age at delivery was 33.8 ± 2.7 weeks in the CIQ group and 37.5 ± 0.7 weeks in the BIQ group. Cesarean delivery was performed in 62.5% of participants, including both BIQ users. The mean birth weight was 2.7 ± 1.1 kg in CIQ and 3.0 ± 0.1 kg in BIQ. Small and large for gestational age were noted in both groups, and one participant’s child had macrosomia. All participants’ children required NICU admission.

**Table 4:** Maternal and neonatal outcomes † Preeclampsia as noted in chart review. ‡ Birth weight not available in delivery records for two CIQ participants. NICU: neonatal intensive care unit; CIQ: control IQ; BIQ: basal IQ; SD: standard deviation

Characteristic	Overall (n=8)	CIQ (n=6)	BIQ (n=2)
Early delivery <37 weeks	4 (50%)	4 (67%)	0 (0%)
Gestational age at delivery (weeks) - average ± SD	34.8 ± 2.9	33.8 ± 2.7	37.5 ± 0.7
Mode of delivery			
Cesarean section	5 (62.5%)	3 (50%)	2 (100%)
Vaginal	3 (37.5%)	3 (50%)	0 (0%)
Preeclampsia^†^	4 (50%)	3 (50%)	1 (50%)
NICU stay >24 hours	8 (100%)	6 (100%)	2 (100%)
Small for gestational age	1 (13%)	1 (17%)	0 (0%)
Large for gestational age	3 (38%)	3 (50%)	0 (0%)
Mean birth weight (kilograms) - average ± SD^‡^	2.8 ± 0.9	2.7 ± 1.1	3.0 ± 0.1

## Discussion

This study of the Tandem t:slim X2 insulin pump illustrates challenges in the care of pregnant individuals with T1D using AID technology that was not originally designed for pregnancy. Even though frequent and numerous pump setting changes were made, participants paradoxically had increasing TAR and TBR and decreasing TIR by the third trimester. CIQ participants tended to have a higher TAR, mean sensor glucose, and A1C compared to BIQ users. Conversely, BIQ users had a higher TBR.

Participants with a greater proportion of bolus insulin usage had a higher TIR. This is in line with other studies showing a greater increase in mealtime insulin needs compared to basal insulin during pregnancy [25]. Further research is needed to determine if there is an optimal basal-to-bolus insulin ratio in each trimester of pregnancy for patients using AID pumps. Bolus timing and hypoglycemia overcorrection can also significantly affect glycemic control in AID users; however, they were not examined in this study [26-28]. Additionally, we have not examined carbohydrate intake due to being unable to verify whether entries were accurate or reflected “fake carbohydrate” boluses, a strategy previously described to lower blood glucose to pregnancy glycemic targets [8].

SAP use and daytime use of sleep activity were strategies seen in some participants to target lower glucose than the Tandem t:slim X2 insulin pump's target range of 112.5 to 160 mg/dL, which is higher than recommended in pregnancy. While these may be helpful techniques for select patients, they need to be balanced against the need to administer manual correction boluses. If using SAP, greater vigilance is also required to prevent and treat hypoglycemia.

Of note, the Medtronic 780G insulin pump has the lowest currently available glucose target of 100 mg/dL. While this pump has been used in several countries, it has only recently been approved in the United States [29,30]. Munda et al. demonstrated a third trimester TIR of 83.6% with the use of this pump in pregnancy [10]. This TIR is substantially higher than what was seen in our study, as well as other studies of HCL pumps in pregnant individuals. This may be due to the MiniMed 780G pump’s lower glucose target. There is a pressing need for pump manufacturers to make lower glucose targets for individuals, such as pregnant patients, who would benefit from this feature.

For several participants, especially the two BIQ users, regular weekly pump reviews and CGM data were unable to be consistently obtained. As a result, fewer changes were made to pump settings compared to those that shared data regularly. One of the two BIQ participants was lost to follow-up for diabetes care partway through the third trimester and only had one viewable CGM report during this time, due to a lack of computer access and not having a smartphone compatible with the t:connect app.

These differences in care engagement highlight significant challenges in optimizing the care of pregnant individuals with diabetes. As a high-risk population, pregnant individuals with T1D typically have more frequent obstetric visits compared to those without diabetes. As a result, they may have difficulty finding time and transportation for additional appointments for diabetes. Technological difficulties and variable access to computers and smartphones have also contributed to the disparities seen in our cohort. There were multiple participants who could only connect to the clinic Dexcom Clarity account or to the clinic t:connect account, but not both. Differences in glycemic control may, in part, be explained by these differences in care participation and opportunities for providers to optimize settings.

Despite a relatively low average pre-pregnancy A1C, adverse pregnancy outcomes were frequent in this cohort. This may be explained in part by the difficulties in achieving the recommended pregnancy glycemic goals. Half of all participants delivered prematurely, with the mean gestational age notably 33.8 weeks in the CIQ group. Cesarean sections were performed in 62.5% of participants, and NICU admission was required in all participants’ children. These statistics are comparable to population-based studies of pregnancies complicated by T1D [26]. Our participants did have a 50% higher incidence of preeclampsia. An elevated pre-pregnancy BMI may have been a contributing factor.

Our study had several limitations, including the small sample size from a single center. We additionally excluded several potential participants for reasons including not being connected to clinic remote monitoring accounts and delivering at hospitals without shared EMR. BIQ users are underrepresented and provide less CGM and insulin pump data compared to CIQ participants. Multiple participants were unable to provide data on a weekly basis, and some only remotely shared insulin pump or CGM data rather than both. Some participants additionally did not establish care until late in the first trimester, contributing to gaps in the data.

Behavioral factors such as bolus timing, hypoglycemia correction, and carbohydrate entry were unable to be analyzed. Participants were seen in two different specialty clinics, and we were unable to delineate the differences in pump education and staff experience with troubleshooting technological difficulties at each site. Additionally, we were unable to examine outcomes such as neonatal hypoglycemia due to only being able to access maternal EMR.

Strengths of our study include calculating and reporting pregnancy goal-specific glycemic ranges. We have additionally reported variables that have not been formally studied, such as duration of sleep activity and SAP use, as factors influencing glycemic control in pregnancy. We have also reported the relationship between basal-to-bolus insulin usage and time in range in our cohort. To our knowledge, this is also the first case series to compare the use of BIQ and CIQ technologies in pregnant individuals with T1D. This exemplifies the need for future research on emerging AID technologies and their use in pregnancy.

## Conclusions

This study describes eight pregnancies in individuals with T1D using the Tandem t:slim X2 insulin pump. Larger-scale studies are needed to understand the differences in CIQ and BIQ efficacy and strategies to adapt these technologies for use in pregnancy. As AID technology becomes more common, its use in pregnancy is also increasing. Therefore, it is imperative for device manufacturers to develop methods to lower target glucose to the recommended pregnancy ranges. Until these advances are made, practitioners must understand the details of insulin pump algorithms to provide individualized care for pregnant patients with T1D.
